# Developing person-centred leadership practices: Health and social care leaders’ experiences following an educational programme

**DOI:** 10.1371/journal.pone.0354356

**Published:** 2026-07-22

**Authors:** Charlotte Klinga, Qarin Lood, Eric Carlström, Emmelie Barenfeld

**Affiliations:** 1 Department of Learning, Informatics, Management and Ethics, Medical Management Centre, Karolinska Institutet, Stockholm, Sweden; 2 Centre for Person‑Centred Care (GPCC), University of Gothenburg, Gothenburg, Sweden; 3 Department of Health and Rehabilitation, Institute of Neuroscience and Physiology, Sahlgrenska Academy, University of Gothenburg, Gothenburg, Sweden; 4 Department of Learning and Leadership for Health Care Professionals, Institute of Health and Care Sciences, Sahlgrenska Academy, University of Gothenburg, Gothenburg, Sweden; Case Western Reserve University, UNITED STATES OF AMERICA

## Abstract

**Objective:**

Educational programmes aimed at nurturing person-centred leadership are requested to advance the implementation of person-centred care, however there is a scarcity of evaluations. This study aimed to explore health and social care leaders’ experiences of changes in leadership practices following participation in an educational programme on person-centred leadership.

**Design:**

A qualitative exploratory research approach, with individual interviews analysed using conventional content analysis, including an interpretative step linking categories to an overarching theme using three leadership concepts.

**Setting:**

Health and social care services in Sweden.

**Participants:**

Thirteen leaders were purposefully selected according to sampling criteria.

**Results:**

The programme did, according to the participants, contribute to the development of person-centred leadership practices by enhancing self-leadership, team leadership, and workforce development aligned with person-centred principles. Three categories unfolded this process: *Live as you learn*, *Strive for equal relations*, and *Enable co-creation*, which were further detailed into nine sub-categories. The results were influenced by various preconditions for continuous learning.

**Conclusion:**

The findings emphasise the necessity of establishing preconditions promoting person-centred leadership (e.g., continuous support from other leaders, time to coach teams and establishing empowering working methods) for continuous development during and after the educational programme. When these preconditions were met, the programme was experienced to effectively influence self-leadership, enhance team leadership, and promote workforce development in line with person-centred principles.

**Implications:**

It is crucial to integrate practical assignments into workplace settings, address daily challenges, and involve leaders, employees, patients, and their relatives early on. This approach supports the continuous development of leadership practices and ensures the sustainable transformation of health and social care practices towards person-centred care. The programme’s theoretical foundation was considered applicable in supporting leadership development, but more research is needed to evaluate the impact and effect of person-centred leadership programmes over time from leaders’, employees’, and patients’ perspectives.

## Introduction

Person-centred care (PCC) is promoted to enhance patient involvement and efficiently utilise healthcare resources [1,2]. This approach aligns with the ongoing global transition towards person-centred and integrated care [2], which underscores a need for leaders in health and social care to align their leadership practices with the evolving paradigm of PCC [3]. This need is further driven by the complexities of care delivery and the imperative for improved patient outcomes [4].

PCC involves a collaborative approach that emphasises the integration of patients’ and professionals’ knowledge and experiences in the co-creation of care [5]. ‘Co-creation’ refers to a process of creative problem-solving involving diverse groups of people throughout all stages of an initiative, from identifying problems and generating solutions to implementation and evaluation [6]. Grounded in an ethical framework that highlights striving for a meaningful life in relation to others, supported by just organisational structures [7, 8], PCC highlights the importance of co-creation through collaboration between health and social care professionals and patients to determine what truly matters to the patients. This approach integrates patients’ resources, needs, and responsibilities with the clinical assessments, professional experience, and evidence-based knowledge of professionals. Through this partnership, care decisions are made in alignment with what is meaningful to the patient, while maintaining ethical principles of justice, mutual respect, and shared responsibility [7,9].

In Sweden and various other European nations, policies and governance frameworks actively promote PCC [10–12]. However, despite these policy endorsements, the practical implementation of PCC remains insufficient [13,14]. Effective leadership is essential for driving change initiatives such as the adoption of PCC. Leaders play a pivotal role in creating the conditions necessary for PCC to flourish, including fostering organisational cultures that support learning and reflection on person-centred ethics [13,15,16]. In practice, this means that leaders in health and social care are expected to model behaviours and structures that promote shared decision-making and collaboration among leaders, care professionals, and patients [7,17]. Moreover, leaders are responsible for supporting and encouraging professionals to develop the competencies and attitudes required to practise PCC effectively [18]. This includes guiding efforts towards PCC, utilising tools that facilitate co-creation, and maintaining a focus on developing innovative working methods from a bottom-up perspective [19]. Balancing top-down directives with bottom-up perspectives in accordance with a PCC approach introduces new demands on leadership and challenges traditional hierarchical models of care [20].

Previous research underscores the significance of person-centred leadership in integrating PCC into everyday practice [17,21]. In this study, person-centred leadership is defined as a relational and dynamic approach to leadership that empowers both employees and leaders by promoting trust, responsibility, innovation, and collaboration. A leader who adopts a person-centred leadership style empowers employees to realise their potential while working towards a shared vision or common goal [21]. This perspective extends the ethical foundations of PCC to the organisational level, where leadership strategies, routines, and practices are defined [7,21,22]. Given the pivotal role leaders play in facilitating the transition to PCC, there is a clear need for interventions that cultivate a broad range of leadership competencies. These include not only interpersonal and intrinsic skills but also the capacity for critical reflexivity [23]. Despite this need, few educational programmes targeting health and social care leaders have specifically focused on developing person-centred leadership to support the implementation of PCC [15].

Existing studies on educational programmes on person-centred leadership have reported improvements in leadership skills [24,25]. Additionally, a participatory action research study describes key attributes, relational processes, and contextual factors that may influence person-centred leadership practices [21]. However, there remains a gap in understanding of how educational programmes may either facilitate or hinder the integration of person-centred leadership in both health and social care settings. Furthermore, leaders’ experiences of person-centred leadership practice after participating in educational programmes need further exploration. To address this gap, this study aimed to explore health and social care leaders’ experiences of changes in leadership practices following participation in an educational programme on person-centred leadership. Details of the educational programme are described in the following methods section.

## Materials and methods

### Study setting

#### Health and social care context.

The study was conducted within the Swedish health and social care system, which is currently undergoing transformation towards more person-centred and integrated care across health and social care settings [26,27]. Swedish health and social care is characterised by a decentralised governance structure where regions are responsible for medical and specialised whereas municipalities are responsible for health and social care in people’s homes [28]. This multi-level governance system requires coordination and collaboration across organisational and professional boundaries to achieve person-centred and integrated care. The leadership programme under study was developed to support the ongoing transformation.

#### The educational programme.

The University of Gothenburg Centre for Person-Centred Care (GPCC) and the Swedish Association of Health Professionals (SAHP), i.e., the main trade union for registered nurses, midwives, radiographers, and biomedical scientists in Sweden, collaborate to deliver a six-month educational programme on person-centred leadership [15]. The programme aims to strengthen leaders’ capacity to promote and implement PCC, with a particular focus on leading and managing change processes in complex organisational contexts. It is offered to SAHP members in leadership roles, including development leaders and managers, working across a range of health and social care settings and serving diverse patient groups. SAHP members were chosen as the target group because they constitute a large proportion of middle managers in Swedish health and social care. These leaders are typically responsible for operational activities and work in close proximity to both healthcare professionals and patients. This group was therefore considered to be particularly well positioned to translate person-centred leadership into practice and thereby contribute substantially to the ongoing national transformation towards person-centred and integrated care. During the study period (2022), 80 leaders were admitted to the programme.

The programme comprised six modules covering key aspects of PCC, leadership, and ethics:

1. Foundations of PCC and person-centred leadership
2. Communication and narration: self in relation to others
3. Strategies for implementing PCC
4. Being person-centred and leading with a person-centred approach
5. Ethical dilemmas and legal jurisdictions relevant to PCC
6. Leading future care: presentation of developmental projects

A blended learning format with both digital, in-person and workplace-based learning activities was applied. The pedagogical approach was based on a flipped classroom method [29], drawing on experiences from a higher education course in PCC to stimulate learning based on person-centred principles [30]. Each module included individual preparation followed by a workshop session to promote reflective practices and experiential learning. Preparatory activities included reading course literature [31], watching two to three recorded lectures (each film 20–30 min long), and completing practical homework assignments in the workplace. These homework assignments encouraged collaboration with employees, patients, and peers. Participants also maintained reflective journals to document insights and questions, which were later discussed in the workshops. The workshops combined full-group discussions with smaller peer-support groups consisting of four to six programme participants. These activities were facilitated by a team of two management strategists and two researchers (the first and last authors). The programme’s learning activities were designed to support the development of two implementation plans: one focused on person-centred leadership and the other on PCC. The pedagogical approach emphasised initiating both plans during the programme while recognising implementation planning as an ongoing process that would continue beyond its completion. Before the programme concluded, participants selected one of the plans for further development and submitted it as their final examination assignment [15].

In the programme curriculum, various aspects of leadership, such as communication, improvement science, and team development, were combined with the GPCC’s framework on PCC. This framework comprises three routines: 1) *initiating a partnership* – using narratives as tools, 2) *working in partnership* – applying shared decision-making on ways forward, and 3) *safeguarding the partnership* – sharing documentation of narratives and joint decisions among involved partners [32]. In the educational programme, all learning activities were designed to integrate these three routines to support leaders in translating person-centred ethics into leadership and care practices [15]. Specifically, each activity incorporated elements of narration, shared decision-making, and shared documentation–such as collages, written summaries of agreements, and implementation plans–to illustrate how the framework could serve as a practical tool for implementing PCC in leadership practices. Further details on the programme are provided by Lood et al. [15].

### Participants

After the programme ended, participants were purposefully selected for interviews to achieve a heterogeneous sample concerning the following pre-defined criteria: leadership experience, leadership roles in PCC implementation, number of employees, level of care, and geographical location. The aim was to ensure that the sample reflected the diversity of leaders who had participated in the programme. To be eligible, participants should have completed the programme in 2022 and consented to participate in the larger research project of which this study formed a part. Contact details to eligible persons were provided by the SAHP. Participants were recruited continuously from mid-January to mid-March 2023 until information power was considered to have been reached [33]. In total, 13 persons were invited to participate by the first author via telephone, and all agreed to participate in an individual interview.

Demographic details on gender, age, leadership role and experience, number of employees, setting, and geographical location are provided in Table 1. Participants’ leadership experience ranged from 3 to 20 years, with most having held their current leadership role for at least three years. Four participants had primary responsibility for the overall implementation and compliance of PCC at higher organisational levels, while nine were actively involved in leading implementation efforts within their respective units or workgroups. The participants represented different regions across Sweden and worked in various health and social care sectors, including specialised care, regional primary care, prehospital care, social care, and school health services.

**Table 1:** Demographic details of participants.

Characteristics	n = 13
**Women/Men/Other**	11/2/0
**Age in years;** Median (Range)	54 (40-60)
**Leadership role**	
Manager	8
Leader of development work	5
**Years at current leadership position**	
1-2	1
3-5	7
6-10	5
**Year of leadership experience;** Median (Range)	7 (3-20)
**Number of employees;** Range	0-74
**Care setting**	
Hospital-based specialist care	3
Regional primary care or prehospital care	4
Social or school health services	6
**Geographical location**	
Northen Sweden	3
Central Sweden	6
Southern Sweden	4

### Data collection

Individual interviews were conducted via digital face-to-face meetings between 27/01/2023 and 16/03/2023, which was three to five months after the end of the educational programme. All interviews were conducted by the first author and lasted 40–65 minutes (mean: 52 min). A question guide was used (see S1 File), which aimed to capture how participation in the programme had influenced leadership practices in the health and social care sectors. The interviews began with an open-ended question aiming to capture the starting point of the learning process on person-centred leadership: ‘Can you please tell me what it was that made you apply to the leadership programme?’ This question was followed by predefined questions in areas focusing on how the programme had influenced the participants’ understanding, knowledge, and practical application of person-centred care and leadership. Participants were prompted to share and illustrate their experiences, highlighting tangible situations and events that had influenced their leadership practices both during and following their involvement in the programme. All interviews were audio recorded and transcribed verbatim for analysis.

### Data analysis

The interviews were analysed using conventional content analysis as described by Hsieh and Shannon [34]. The first and last authors independently examined the transcriptions to gain a comprehensive understanding of the data and identify segments relevant to the research objectives. Initial impressions were documented by the first author to guide the preliminary analysis. The first author then led the coding process, assigning descriptive labels to text units that closely reflected their content. This stage involved an abductive approach, where the interpretation of codes was informed by the first author’s pre-understanding as a researcher in leadership and organisation of health and social care. Based on this preliminary understanding and an examination of the literature, three analytical tracks (self-leadership, team leadership, and workforce development) were applied to structure the interpretation, moving from the individual leader to the workforce through team leadership. *Self leadership* comes first, before leading others. It is the practice of self-influence directing one’s own thoughts and actions. Self leadership requires self-awareness, goal orientation, and openness to external influences to achieve desired outcomes [35]. *Team leadership* is the process of building a cohesive unit, motivating team members, and supporting their professional growth. Additionally, it involves guiding a group of people towards a common goal, fostering collaboration, and ensuring that each member contributes their best [36]. *Workforce development* is a proactive and future-oriented approach to ensuring that employees can perform their roles effectively, adapt to changing workplace needs, and contribute to organisational success. It also includes strategies and initiatives aimed at enhancing the skills, knowledge, and abilities of employees to meet upcoming job demands [37]. Based on this pre-understanding, three analytical tracks were applied to structure the interpretation: self-leadership, team-leadership, and workforce development. These tracks helped frame emerging patterns in the data. In the next phase, codes with similar meanings were grouped into categories and sub-categories, which were then interpreted at a latent level through iterative clustering and refinement.

### Establishing trustworthiness

Trustworthiness was established in accordance with recognised criteria for qualitative research: credibility, dependability, confirmability, reflexivity, and transferability [38]. Credibility was strengthened through an iterative and collaborative analytic process. Coding and categorisation were conducted jointly by the first and the last authors using a negotiated consensus as described by Bradley et al. [39]. Regular discussions among all authors were held throughout the analysis to refine interpretations and reach agreement on the final categories and sub-categories, culminating in an overarching theme. To further enhance credibility, two authors (QL, EC) who had not been involved in the educational programme contributed external perspectives, enabling critical examination of emerging interpretations through peer debriefing.

Dependability and confirmability were supported by a transparent and systematic analytic process. An audit trail was maintained by documenting the progression from meaning units to sub-categories, categories, and theme, as illustrated in Table A S1 Appendix. Reflexivity was addressed throughout the study through ongoing reflection on how the researchers’ backgrounds, experiences, and pre-understandings might influence data collection, analysis, and interpretation. Particular attention was paid to the involvement of two authors (CK, EB) in the educational programme. The research team comprised female (CK, QL, EB) and male (EC) researchers from diverse professional backgrounds, including social work, occupational therapy, and nursing, with expertise in person-centred and integrated care, qualitative research, higher education (CK, QL, EB, EC), and management (CK, EC). Consideration of how these characteristics may have shaped the analytic process formed an integral part of the reflexive work.

Transferability was facilitated through detailed descriptions of the Swedish health and social care setting and the educational programme, together with the inclusion of illustrative quotations that demonstrate the connection between the data and the study findings [40]. To improve readability, quotations were translated from Swedish into English with minor linguistic adjustments; omissions are indicated by […].

Finally, to enhance transparency and completeness of reporting, the study was conducted and reported in accordance with the Consolidated Criteria for Reporting Qualitative Research (COREQ) [41].

### Ethical approval and informed consent

The Swedish Ethical Review Board granted approval for this research project (Dnr 2022-04052-01). Participants were informed, both verbally and in writing, that their involvement was entirely voluntary and that they could opt out of the study at any time. Written informed consent was obtained from all participants.

## Results

**Fig 1:**
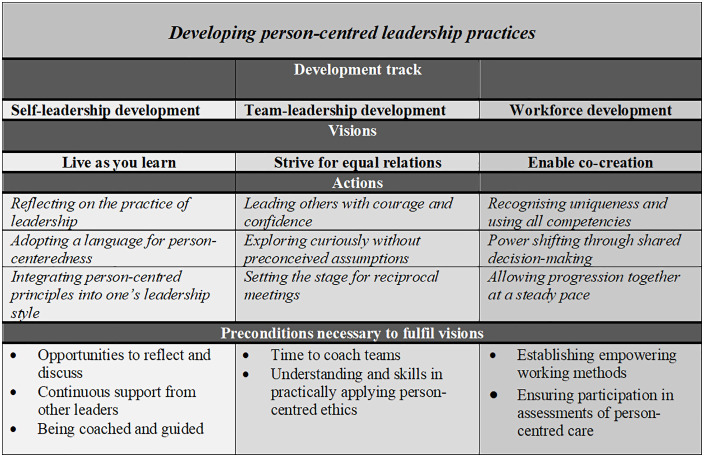
Overview of the results.

The results illustrate how participation in the educational programme was experienced to influence leadership practices in health and social care. The overarching theme, *Developing person-centred leadership practices,* captures the transformative process participants underwent as they translated person-centred ethics into practical leadership actions. This development process varied between participants and was described across three distinct yet interrelated development tracks (self-leadership, team-leadership, and workforce development), each aligned with a leadership vision represented by the categories: *Live as you learn, Strive for equal relations,* and *Enable co-creation.* Actions aimed at realising these visions are organised into nine sub-categories. Additionally, the specific preconditions necessary to fulfil each vision are outlined within the text under each category. For an overview of the results, see Fig 1.

### Live as you learn

The vision *Live as you learn* symbolises how the programme influenced participants’ personal journeys in developing self-leadership by encouraging them to translate their values into actions. This was reflected in participants’ experiences of having opportunities to align their personal growth with person-centred ethics. Key preconditions for realising this vision included continuous support, both through encouragement and input from their own managers and from fellow leaders. Participants expressed a need for opportunities to discuss questions, challenges, and experiences, and to engage in reflective dialogues with peers. These exchanges helped them navigate complex issues, share insights, and broaden their perspectives. Additionally, access to coaching and guidance was seen as essential. Three sub-categories outline the actions participants took to cultivate self-leadership in line with the vision: *Reflecting on the practice of leadership; Adopting a language for person-centeredness;* and *Integrating person-centred principles into one’s leadership style*.

#### Reflecting on the practice of leadership.

Participants expressed that having the opportunity to contemplate their own leadership in the context of person-centred principles was both fulfilling and beneficial for their self-leadership development. In the fast-paced environment of health and social care, such opportunities for self-reflection were rare and the programme created space for participants to assess and review their leadership approach and their capacity to lead in a person-centred way. Self-reflection was also described as essential for recognising that others may perceive situations differently, underscoring the importance of examining one’s own assumptions and perspectives to improve interactions with others. As one participant articulated:

*You get the chance to pause and reflect in a way that you don’t usually do. And to reflect on precisely this: How am I as a leader? Am I person-centred? Do I see my employees? Am I working to get to know them? (Interview 10)*

Throughout the programme, participants immersed themselves in profound reflection on their leadership practices, particularly in relation to person-centred ethics. This process, anchored in self-leadership, combined individual reflection with peer dialogue. Through various reflective exercises, they were prompted to critically examine their roles, responsibilities, limitations, and strengths. These exercises were both challenging and empowering, leading to meaningful insights that supported self-leadership development. One participant shared their thoughts on the impact of this reflective process:

*It’s as if I’ve reflected in sync and worked on myself, associating things that we’ve had in the training. Bringing them together with my organisation and connecting them. What I’ve learned, how it is in reality, and how I work. (Interview 1)*

#### Adopting a language for person-centeredness.

Participants experienced notable enhancements in their ability to communicate the principles of PCC to other leaders and employees. They recognised that interpretations of PCC and person-centred leadership can vary considerably, and that clearly articulating their own understanding was essential for effective leadership. Through the programme, their self-leadership was developed through greater confidence and precision in expressing their perspectives, acknowledging that defining key concepts was a vital part of advancing as person-centred leaders. As one participant noted:

*It’s about using words without defining what we mean by those words. So, when we say, ‘person-centred leadership’ or ‘person-centeredness’, before doing much more and working together, we need to define what we mean here in this context. It became very clear that we had somewhat different perspectives on it. (Interview 5)*

Another participant highlighted that they regularly shared insights from the programme with their employees and involved them in assignments between sessions. This helped facilitate the adoption of a shared language for person-centredness in practice and demonstrated a proactive approach to self-leadership:

*So, I thought like this, ‘Now, let’s incorporate a person-centred approach in a small, at least in a small part, of this big project.’ [...] I’ve talked about person-centred care at almost every monthly team meeting and [about] what I had learned from the training. They got to learn from me each time I learned something new. (Interview 11)*

#### Integrating person-centred principles into one’s leadership style.

Throughout the programme, participants experienced a transformation in mindset, reshaping their understanding of leadership, moving towards a more person-centred approach. This transformation was an important aspect of their self-leadership development, as it involved critical reflection on their own values, behaviours, and assumptions. By dismantling internal barriers and broadening their perspectives, participants were able to develop innovative solutions to complex challenges in their leadership practice. The programme ignited a profound understanding of, and appreciation for, employees as persons, reinforcing the importance of becoming more person-centred leaders, as one participant expressed:

*That would probably be it, that I try more and more to think about who I have in front of me. It facilitates things. It also helps privately sometimes when one tries to understand. ‘Why does this happen or...?’ (Interview 12)*

As described by the participants, the programme further provided them with valuable opportunities to apply their knowledge in real-world situations, which promoted both personal and professional growth. Even those with significant experience in PCC found the programme to be both challenging and affirming. Engaging in reflective processes was central to their self-leadership journey, as it encouraged them to critically assess their leadership practices and previous assumptions. As one participant described:

*Now, you have really been able to challenge yourself in certain things, and some things may have been a confirmation of what you already know. […] I feel that, yes, we have been in this, although we haven’t thought about it as systematically as we do today, and neither have I. […] I feel that I have received a lot of support in the programme because I didn’t quite think like that before. (Interview 10)*

### Strive for equal relations

This vision shed light on the impact of the educational programme on the development of team-leadership, particularly in terms of shifts in leadership behaviours and relational dynamics with teams of employees and other leaders, as well as patients and their relatives. The programme was experienced to enhance participants’ ability to lead and support teams in a *Strive for equal relations,* promoting inclusive and empowering interactions. Key preconditions for realising this vision included having sufficient time and opportunities to coach teams on motivation, shared purpose, and goals related to PCC in an ever-changing environment. It also required a solid understanding of person-centred principles, the ability to apply them in practice, and a commitment to modelling these values during the transition towards PCC. To develop team-leadership aligned with this vision, participants described how they engaged in three actions described in the sub-categories: *Leading others with courage and confidence, Exploring curiously without preconceived assumptions,* and *Setting the stage for reciprocal meetings*.

#### Leading others with courage and confidence.

Participants emphasised how the educational programme significantly strengthened their skills to lead their teams. They felt empowered to create space for others by adopting a person-centred approach to both leadership and care. This shift was underpinned by a growing sense of confidence and clarity in their roles as leaders of their teams. They described a transformation in their leadership style, from previously uncertain to more grounded and assured, thanks to the programme. One of the participants expressed their empowerment as follows:

*I felt empowered in the sense that I’m doing the right thing. I’m on the right track. I have the feeling that if I hadn’t taken that leadership training, maybe I would have faltered along the way, quite simply. (Interview 8)*

Participants also shared a revitalised sense of self-assurance and stability in leading their teams. By integrating new tools and perspectives, they experienced a deeper sense of calm and control. Although some had previously doubted their team-leadership abilities, they now felt the courage to recognise and acknowledge their strengths. The programme equipped them with a fresh perspective on how to empower their teams, promoting a more inclusive, collaborative, and confident leadership environment. As one participant articulated:

*Now I have a few more tools in my little toolbox that I can use, and I don’t get so nervous or worried if I find myself in situations where I feel like, ‘No, I don’t know. I have no solution for this today.’ Without knowing how to act. I might not have an answer, but I’ll come back. I believe that the training, combined with other factors, has made me feel more confident as a manager, absolutely. (Interview 4)*

#### Exploring curiously without preconceived assumptions.

Participants noted that one of the most significant changes they experienced following the programme was the development of a more curious and open-minded approach to team-leadership. This shift in mindset marked an essential counterbalance to their prior practices, where they frequently assumed they understood the needs of employees, patients, and relatives. Such assumptions had previously limited their ability to lead in a truly person-centred way. Through the programme, participants learned to cultivate curiosity as a core team-leadership skill, asking questions rather than relying on assumptions. This change enabled them to better understand the unique perspectives and needs of those they lead. One participant reflected:

*I often say it is intriguing that we, as care professionals, think we know what patients and relatives need and have a pretty good idea about it. But when you speak to patients and relatives, you see that... sure, some things are entirely correct, absolutely. But there’s also so much that is individual-dependent, which is not at all what we assume. (Interview 5)*

Participants also found it challenging yet immensely rewarding to engage in conversations without steering them too much. This required them to listen with full attention and to suspend their own preconceived judgements and expectations. The quotation below highlights how they experienced active listening as a both demanding and enlightening practice that deepened their ability to lead with presence.

*And then I tried to let them just speak for themselves. I find it difficult because it’s easy for me to... that I want to try to control it [...] You also got to hear what they do, things you don’t see when they’re working. They were supposed to give examples of when they worked in a person-centred way, and it was great because a lot of things came up, things they do here on weekends when I’m not here, and so on, so it was fun. But maybe I should dare to let them speak even more instead of trying to steer a little. (Interview 12)*

#### Setting the stage for reciprocal meetings.

During the programme, participants described how they deepened their understanding of their own leadership strengths by actively engaging employees in tasks and discussions related to PCC. A key area of team-leadership development was their ability to foster more meaningful and reciprocal interactions with their teams. They recognised the importance of being more prepared to invite employees to scheduled meetings and of being fully present during conversations, as highlighted in the quotation below.

*I’ve thought about many things there that I’ve changed or tried to work on, especially my presence in a meeting. How I’m present when I meet my employees and other people. In hindsight, I’ve reflected that I was often maybe somewhere else or far ahead in my thoughts. Now I try to be more present in the meeting. (Interview 1)*

Participants further expressed that they uncovered valuable insights by altering their approach to conversations and meetings. They gained deeper insights into both operational dynamics and employee well-being. Engaging with employees in their daily work, prior to performance reviews, allowed for more grounded and informed discussions. Scheduled meetings became opportunities to tackle important topics in more innovative and impactful ways. Moreover, many participants observed that they had become better at encouraging others to actively engage in conversations. This shift towards a more inclusive dialogue came about through reflection and growth during the programme, as illustrated in the following quotation:

*This exercise allowed me to reflect on how I invite people to conversations. What are the effects depending on how you organise the conversation or how you invite someone? [...] it gave me insight into what I should do to get sincere answers and a sense of how they think and [what they] believe, and so on. Not to be too authoritarian, perhaps. (Interview 7)*

### Enable co-creation

Guided by the vision *Enable co-creation,* the educational programme influenced participants’ efforts to involve employees in workforce development. In this context, workforce development referred to efforts aimed at engaging employees in transforming care processes, workplace culture, and the use of resources, always aligned with person-centred ethics.  They also identified several critical preconditions for genuine workforce development in co-creation. Preconditions that were not yet fully realised in practice. Notably, they stressed the need for greater involvement of patients and relatives in evaluating whether care had truly become more person-centred. Without such involvement, they argued, authentic co-creation could not be achieved. Furthermore, participants underscored the importance of developing working methods that empower both employees and patients to actively engage in assessments of the person-centredness of care. This includes ensuring that their voices are heard, and their influence is recognised. Actions supporting participants’ workforce development are described in three sub-categories: *Recognising uniqueness and using all competencies; Power shifting through shared decision-making;* and *Allowing progression together at a steady pace.*

#### Recognising uniqueness and using all competencies.

As part of their workforce development efforts during the programme, participants recognised the significance of treating employees with the same level of care and respect as patients and their relatives. This shift in perspective was key to cultivate a more positive and productive workplace culture. By adopting a person-centred approach to leadership, participants described that they felt empowered to build successful teams, grounded in open communication and mutual trust between leaders and employees. A vital component of such workforce development was the recognition of each employee’s unique strengths and areas for growth. The practical exercises within the programme encouraged participants to regularly evaluate and address employees’ needs, ensuring that everyone could effectively leverage their skills in the workplace. As one participant noted:

*Maybe it’s more about me thinking that way, trying to see nurses also as individuals in a different way, and actually trying to consider how their skills fit into the right context and that we are good at different things, and it’s important to incorporate everyone’s skills. I think I try more to engage in broader dialogues, perhaps more openly than I did before. But it’s not certain that it’s visible. (Interview 11)*

As participants began to organise and apply their own and employees’ competencies in a more organised manner, they noticed a broader range of perspectives emerging in discussions. As the programme advanced, this led to increased employee engagement in developing the workplace. Participants realised that effective leadership does not require them having all the answers. Instead, they found it beneficial to systematically involve others early in the process, co-creating solutions with employees. As described by one participant:

*Can we look at it together and see how we can improve each other’s skills? That’s also a part of it, not thinking that one must know everything, but that this is a way we could make it clearer. (Interview 13)*

#### Power shifting through shared decision-making.

Participants shared their insights on how the programme supported a shift in their leadership approach by promoting shared decision-making as an element of workforce development. By involving employees earlier in operational decisions, participants began to feel that the workplace was more inclusive and collaborative, helping them to feel less isolated, and making the workplace feel more enjoyable. However, participants observed that altering established working methods and enhancing employee involvement in decision-making was a lengthy and challenging endeavour. It required practice and a willingness to let go of control, as exemplified by one participant:

*I may have an idea, but I don’t necessarily need to present my idea; instead, I can ask, ‘Do you have any ideas on how we can solve this?’ And that’s something I’ve had to practise a lot. I am responsible for the decision, but I don’t need to make the decision without my employees being able to contribute with their own ideas. I can say, ‘If you think this is the best solution, I’ll accept it.’ (Interview 4)*

Participants also noted that employees often felt uncertain about assuming responsibility, prompting them to reconsider their previous, more controlling, approaches to leadership. The programme helped them develop the ability to temper their own need for control, listen more actively and create space for dialogue between them and employees. This promoted collaborative thinking rather than leaders making decisions on behalf of employees, seen as essential for more sustainable workforce development as illustrated by the following quotation:

*It’s about listening more, not thinking that you know best and have all the solutions. I’ve discussed this a lot during the training with my closest colleague, who is also very clear and direct... but we have actually changed... we have reflected and changed our approach, to listen more, to have more of this dialogue, to listen. (Interview 2)*

#### Allowing progression together at a steady pace.

Participants found that adopting a person-centred approach to their leadership during the programme helped them to achieve a joint and steady development towards PCC at their units. They reflected on the importance of understanding their own operations, the needs of employees, and the shared purpose of their work. They also gained tools for change management, motivation and communication. Moreover, the programme introduced structured routines for PCC (initiating a partnership, working in partnership, and safeguarding the partnership), which provided a practical framework for leading change collaboratively. These routines helped participants manage change with greater clarity and calm, as one participant described:

*Yes, but I’ve had this mantra, narrative, partnership, documentation. It has become a mantra for me, to say, ‘This is what we should look at and raise our gaze.’ How should one think about it at all levels? (Interview 13)*

Participants also recognised the challenges of adopting new work practices and the significance of setting clear goals. The programme inspired them to reflect on and value the importance of establishing both personal and organisational goals for the progress of PCC, aligning leadership objectives with employee needs and broader organisational aims. They stressed the necessity of anchoring change efforts among all persons involved to ensure a shared understanding and commitment. As one participant stated:

*Yes, that’s something I’ve struggled with a lot, so that it truly becomes the target group and the employees who decide whether it’s person-centred or not. […] It’s about them having influence, feeling that they have influence, and demonstrating that they do in some way. And we need to ask them and invite them in. (Interview 9)*

## Discussion

This study aimed to explore health and social care leaders’ experiences of changes in leadership practices following participation in an educational programme on person-centred leadership. The main findings illustrate how three visions – *Live as you learn, Strive for equal relations,* and *Enable co-creation* – collectively drove the development of person-centred leadership across multiple levels: self-leadership, team-leadership, and workforce development. Even though there was variation in experienced leadership development following participation in the programme, its theoretical foundation and person-centred pedagogical approach were described as integral, particularly when the key preconditions for learning and application were met.

To enhance the transferability of the findings across diverse leadership styles, the empirical insights from the interviews are interpreted through the lens of leadership theories and previous research. Firstly, the description of self-leadership development aligns with the meta-performance model, which posits that professional development initiatives are most impactful when embedded within broader self-leadership frameworks and reinforced by organisational support [42]. Participants described the programme as a catalyst for sustained leadership growth, offering a blend of conceptual knowledge, practical tools, and reflective exercises that enhanced their ability to lead with clarity and confidence, although the extent of perceived development varied among them. Additionally, our findings identified that ongoing support and encouragement from management, and structured opportunities for reflecting on leadership practices were essential in developing self-leadership. These findings are consistent with prior research by Joseph [43], which highlights the importance of senior leadership’s involvement in creating a supportive environment for middle managers. Such an environment not only facilitates professional growth but also strengthens the foundation for person-centred leadership practices.

Previous research by Harari [44] highlights the importance of aligning leadership education with clearly defined outcomes to effectively promote self-leadership. This alignment also supports leaders in embodying the values they promote, thereby cultivating a person-centred workplace culture [45]. From this perspective, the programme under exploration’s foundation in the GPCC framework [7,32], which emphasises initiating, working in, and safeguarding partnerships, provided a structured approach that encouraged leaders to internalise and enact person-centred ethics. The GPCC framework has previously been shown to facilitate person-centred approaches in professional-patient encounters [46]. Our findings add to this theoretical understanding of PCC by demonstrating the framework’s relevance in operationalising person-centred leadership. During and after the programme, participants dedicated time to reflect on how to initiate, work in, and maintain partnerships with employees, patients, and relatives. This allowed them to critically evaluate their previous assumptions and leadership styles in relation to themselves, the teams that they led, and the workplace. Self-reflection in leadership has previously demonstrated benefits, including enhanced well-being, a more positive work attitude, increased commitment, and improved performance among employees [47]. Similarly, McCance and McCormack [40] describe how both self-reflection and shared reflection support the process of ‘*knowing self’*, which is described as a prerequisite for person-centred practice.

Secondly, our findings highlight the development of effective team-leadership as essential for embedding PCC within health and social care systems. As Alharbi et al. [22] argue, ongoing discussions about purpose, goals, teamwork, and power dynamics are critical to navigating the structural changes required for successful implementation of PCC [22]. While previous studies have pinpointed the role of leaders in promoting teamwork [48] and fostering a person-centred workplace culture [45,49], our findings extend this understanding by illustrating how team-leadership was enhanced and translated into practice following the educational programme. A key theme emerging from our data was the progression of team-leadership behaviours that supported the vision *strive for equal relations*, a cornerstone of person-centred leadership as described by participants. Effective team leadership in health and social care requires a nuanced understanding of team dynamics within a shared operational framework. Recognising team-leadership as a central component of the transition towards PCC is thus essential. As Smith et al. [50] argue, leaders need to be both clinically informed and relationally grounded to lead towards person-centred and integrated care. Our findings reinforce this view, showing how leadership programmes can catalyse such transformation by equipping health and social care leaders with practical tools and perspectives needed to lead collaboratively and effectively.

As described by Middleton et al. [51], there are few studies on behavioural changes after leadership programmes. Our findings describe that such programmes may support behavioural change by encouraging participants to prioritise understanding both employee and patient needs and to value curious exploration over assumptions. Participants appeared to develop their maturity and leadership capabilities, with indications of refined leadership styles and deepened connections with employees through practising work in partnership during the programme. As described by Middelton et al. [51] such leadership skills are crucial in various contexts as enhancing listening abilities enables leaders to engage more authentically with diverse viewpoints. These skills also support adapting leadership styles to different circumstances and the unique needs of different persons [52]. Finally, viewing the transition towards PCC through the lens of workforce development suggests that leadership programmes contribute to cultural and behavioural change. The category *Enable co-creation* offers a valuable perspective on how such programmes support the development of person-centred leadership, showcasing the dynamic interplay between leadership and the workplace as a site of continuous learning and transformation. As Kligler et al. [53] emphasise, transforming a care system requires more than simply adding services or retraining staff; it necessitates a holistic systems perspective. This aligns with Bergholtz et al.’s [54] recent observation that change efforts often falter when key interest holders are not engaged from the beginning. Participants in our study reported that the educational programme helped them realise that they did not need to have all the answers. Instead, they learned to involve both employees and patients in shared decision-making and the co-creation of care, thereby redistributing power and fostering inclusivity. This shift from a paternalistic model to one that treats patients as partners is supported by Alharbi et al. [22], who stress the importance of pedagogical skills in enabling such a transformation. Our findings suggest that the programme’s pedagogical design was instrumental in helping participants engage employees and patients more actively, thereby deepening their understanding and enhancing their capacity to empower others. To supplement the self-perceived changes in leadership behaviour identified in this study, quantitative research is needed to examine actual leadership practice, as well as the perspectives of employees, patients, and the wider health or social care organisation.

Building on existing evidence [21,49], our findings reinforce the idea that the workplace is a vital setting for learning person-centred leadership. Embedding leadership development in real-life contexts allows participants to apply theoretical knowledge to practical situations. When combined with peer-support and structured opportunities for reflection, this approach can nurture collaborative learning and support continuous development on PCC and leadership practices. The inclusion of leaders from diverse organisational levels, health and social care settings, and geographical regions across Sweden enhances the transferability of the findings by identifying common developmental tracks that transcend specific organisational contexts and leadership roles. Consistent with Uvhagen et al. [55], our study identified preconditions at multiple organisational levels that support continuous learning. The educational programme was experienced as contributing to varying degrees, to tangible learning processes that integrated person-centred principles into self-leadership, team-leadership, and workforce development, depending on how the preconditions were met. However, while these preconditions were present, they were not always prominent, suggesting a need for further exploration of the contextual factors that influence the success of person-centred leadership initiatives. Finally, as Garbbet’s [56] definition of practice development underscores, transforming care practices and organisational culture requires systematic, rigorous, and facilitated approaches. This aligns closely with modern understandings emphasising the integration of learning and leadership into the everyday fabric of care environments. Future research should evaluate the long-term impact of leadership programmes from multiple interest holder perspectives to inform the design of more effective, context-sensitive interventions that advance both leadership and care.

## Limitations

The qualitative approach allowed us to gather authentic insights directly from participants, grounding our findings in a diverse range of perspectives through conventional content analysis [34]. The characteristics of the participants may, however, have influenced the findings in several ways. Through purposive sampling, we sought variation in professional background, leadership experience, organisational context, and geographic location, resulting in a sample that broadly reflected the diversity of programme participants. Nevertheless, the sample consisted predominantly of women, which may have influenced how the programme was experienced and interpreted, given that gender can shape leadership practices [57]. Participants were also generally experienced leaders in the middle to later stages of their careers. This may have contributed to constructive views of the programme and an openness to organisational change. At the same time, their extensive leadership experience may have limited the expression of more critical perspectives [58]. Furthermore, the inclusion of both managers and development leaders may have influenced the findings, as these roles differ in operational and strategic responsibilities. Development leaders may, for example, be more accustomed to working with organisational development initiatives and policy-related concepts than managers.

The predominantly positive experiences reported in this study may have several explanations.  Completion of the programme was applied as an inclusion criterion, and participants may therefore represent persons who were more engaged with the programme and its context. In addition, all participants had chosen to enrol in a programme focused on person-centred and integrated care, suggesting a pre-existing interest in or commitment to these approaches. This may have introduced a positive bias and limited the expression of more critical or divergent views. The study’s focus on experienced changes may also have directed attention towards positive developments, although participants were equally encouraged to describe situations where no change had occurred. While the selected sample allowed us to identify both barriers to and enablers of person-centred leadership development, including participants who did not complete the programme could have provided alternative or more critical insights. These factors should be considered when interpreting the findings and assessing their transferability.

Researcher involvement may also be considered a limitation. The first and last authors were involved as lecturers in the leadership programme, which may have influenced both data collection and analysis. As described in the Methods section, this was addressed through ongoing reflexive discussions and collaborative analytic procedures. To minimise the risk of social desirability bias, participants were encouraged to share both positive and negative experiences during the interviews. The interviewer adopted a neutral stance and emphasised that there were no right or wrong answers.

## Conclusions

The findings underscore the need to promote preconditions for continuous development during and after the programme. Participants experienced that when preconditions were fulfilled, participation could influence leadership practices by fostering self-leadership, enhancing team-leadership, and promoting workforce development in line with person-centred principles. By addressing development across these three levels, the participants gained tools to transform health and social care practices towards PCC. The programme’s theoretical foundation in the GPCC framework facilitated this development, demonstrating the framework’s applicability in a leadership context. The operationalisation of person-centred leadership was further strengthened by the interplay between practical experience, self-reflection, and joint reflections with other leaders, employees, and patients. Transforming care practices and organisational culture requires structured, evidence-based approaches. This aligns with modern workplace development, which embeds learning and leadership into daily practice. Integrating practical assignments in workplace settings appears to foster early engagement from leaders, employees, patients, and relatives. However, additional research is needed to understand how knowledge can be translated into practice.

## Supporting information

S1 File Supporting information. Question guide.
(DOCX)

S1 Appendix Examples of the analytical process specifying the way from text units to categories including preconditions and the overarching theme. (DOCX)

S1 Checklist QOREC. (DOCX)
