# Supplementary material for: Developing person-centred leadership practices: Health and social care leaders’ experiences following an educational programme
Source: PLoS One. 2026 Jul 22;21(7):e0354356. doi: 10.1371/journal.pone.0354356 (PMC13390939; doi:10.1371/journal.pone.0354356)
Supplement: S1 File — Question guide. (DOCX) [file pone.0354356.s001.docx]

**Opening Question**

*Can you please tell me what made you apply to the leadership program?*

- **Expectations before the training and how it turned out**
  - Views and experiences of person-centred care before the training.
  - Views and experiences of leadership before and after the training.
- **Experiences of the leadership program**
  - Content and events during and after the training of specific significance for development/learning (Within the organization, the program and other contexts).
  - Module 1-6, included assignments, peer-learning, and individual studies.
  - Examples of rewarding and non-rewarding events during the training
  - Opportunities or facilitating factors
  - Hindering circumstances or factors
- **Views and experiences of practicing person-centred care after the training**
- **Views and experiences of practicing leadership after the training**
  - If changes: Can you give examples of what in the training contributed to a changed perspective/understanding or how you do things differently today?
  - **Have you received support from the training (e.g., materials and methods) in implementing person-centred care in your practice? If yes, what support and how have you used it?**
  - Is there any part of the training that you believe particularly contributed to an increased understanding or improved conditions for working in partnership with various stakeholders, such as leaders, patients, relatives, and staff?
  - Have you been able to apply your new knowledge in your workplace in relation to the development plan you created? If so, how?
- **Perceived benefits**
  - Thinking more generally about the benefits of the training, do you believe it can be beneficial? What benefits might it provide for leaders, staff, and patients?
  - **There is an ongoing transition to person-centred and integrated care – do you think this course can contribute to that work in any way? Yes, no, why/how?**

**Additional questions asked at the end of each interview**

- If you were responsible for the leadership program, how would you have wished to develop it further?
- Is there anything else you would like to share that I haven’t asked about?
