# Supplementary material for: Developing person-centred leadership practices: Health and social care leaders’ experiences following an educational programme
Source: PLoS One. 2026 Jul 22;21(7):e0354356. doi: 10.1371/journal.pone.0354356 (PMC13390939; doi:10.1371/journal.pone.0354356)
Supplement: S1 Appendix — (DOCX) [file pone.0354356.s002.docx]

**Table A. Examples of the analytical process specifying the way from text units to categories including preconditions and the overarching theme.**

| \| **Overarching theme**  Developing person-centred leadership practice \| \| \| \| \| \| \| --- \| --- \| --- \| --- \| --- \| --- \| \| **Text unit** \| **Reflective notes** \| **Code** \| **Analytical Track** \| **Subcategory**  ***Actions*** \| **Category**  ***Vision*** \| \| *So, I think it is important to reflect on: ‘What tasks do I have? What are my limitations and my strengths?’ I believe that is very important when entering a training programme like this. (interview 3)* \| Reflecting on own leadership to model person‑centredness \| Modelling person‑centred leadership \| **Self‑leadership development** \| Reflecting on the practice of leadership \| **Live as you learn** \| \| *So, I thought like this, 'Now, let's incorporate a person-centered approach in a small, at least in a small part, of this big project.' [...] I've almost talked about person-centered care at every monthly team-meeting and what I had learned from the training. They got to learn from me after each instance I learned something new. So when we were there trying to concretize this, the groundwork had already been laid in some way. (interview 11)* \| Shared language clarifies leadership values \| Acquiring a person‑centred vocabulary \| Adopting a language for person‑centredness \| \| *It is not an entirely new way for us to work, but rather an approach. And I think that is something I can contribute a lot with — that this is not about starting a new model or changing everything. We need to open up and actually look at how we can work together. Because that is very much part of my role as well: we are supposed to work in teams — occupational therapists, physiotherapists, and nurses. They have worked separately before. […] to put the focus on working in a person‑centred way and seeing the whole picture, so that we can identify a common thread. (interview 13)* \| Applying person‑centred principles in daily leadership \| Embedding person‑centred principles \| Integrating person‑centred principles into one’s leadership style \| \| **Overarching theme**  Developing person-centred leadership practice \| \| \| \| \| \| \| **Text unit** \| **Reflective notes** \| **Code** \| **Analytical Track** \| **Subcategory**  ***Actions*** \| **Category**  ***Vision*** \| \| *I do feel that I’ve gained a different kind of confidence, that I dare to convey the difficult things. […] Yes, I think the employees see a sense of confidence in me as a manager — that I am present and that I make decisions. I don’t shy away from making difficult decisions; I actually make them. (interview 4)* \| Leading with courage despite uncertainty \| Courageous leadership \| **Team‑leadership development** \| Leading others with courage and confidence \| **Strive for equal relations** \| \| *It is probably more this curiosity about others. Yes, I think so. To ask more questions, actually. That is something I have also thought about practising. (interview 6)* \| Approaching situations with curiosity \| Curious exploration \| Exploring curiously without preconceived assumptions \| \| *As a nurse, I have the patients, but as a manager I have the employees — and I actually need to show them the same kind of care for the work to function and for the organisation to run well. […] Because if I don’t listen to my employees, to what they bring and who they are as individuals, well, then I might soon have a sick‑leave case on my hands — and how does that help me? (interview 8)* \| Creating conditions for reciprocal interaction \| Facilitating reciprocal meetings \| Setting the stage for reciprocal meetings \| \| **Overarching theme**  Developing person-centred leadership practice \| \| \| \| \| \| \| **Text unit** \| **Reflective notes** \| **Code** \| **Analytical Track** \| **Subcategory**  ***Actions*** \| **Category**  ***Vision*** \| \| *What do they need in order to make full use of all their abilities at work? […] Because I also feel, as a manager, that after being here for eight years it becomes a bit routine. So that’s why it also felt good partly to do it in a slightly new way, and now also to hear what the employees have thought about it and how it is developing. (interview 6)* \| Recognising and utilising diverse competencies \| Valuing uniqueness \| **Workplace development** \| Recognising uniqueness and using all competencies \| **Enable co-creation** \| \| *It's about listening more, not thinking that you know best and have all the solutions. I've discussed this a lot during the training with my closest colleague, which is also very clear and direct... but we have actually changed... we have reflected and changed our approach, to listen more, to have more of this dialogue, to listen. (interview 2)* \| Shared decision‑making redistributes power \| Power‑sharing \| Power shifting through shared decision‑making \| \| *A major change is probably me as the leader of the department and where we are heading together, and what is starting to happen to us. It’s amazing to see. And it’s thanks to the fact that I have opened up more and changed my way of doing things. […] Somehow this feels more enjoyable. Because now I don’t feel as lonely anymore. That I got to open my eyes to leave my loneliness behind. (interview 1)* \| Progressing together at a steady pace \| Collective pacing \| Allowing progression together at a steady pace \| \| **Text unit** \| **Reflective notes** \| **Code** \| **Analytical  track** \| **Preconditions necessary to fulfil visions** \| \| *But in this organisation it is much easier because I have the support of the management – or really the support of the entire organisation. Now it’s just a matter of working with the employees. That makes it easier for me. (interview 13)* \| Coaching enables practical application \| Guided development \| Self-leadership development \| Being coached and guided \| \| *I also don't think they actually don´t conduct the arrival meetings as we have decided, instead they fall back into their old routines, as they have done before. [...]I don't think they take the time. Somewhere along the way, they fall into this 'yes, but we're in a hurry. We have to do it quickly' instead of taking it easy. (interview 8)* \| Without time to coach the team, person‑centred ethics remain theoretical. \| Need for coaching time \| Team development \| Time to coach teams \| \| *"It is also the case that if they are not involved from the start, I think it becomes much more difficult to get them willing to drive the change. If you are involved and able to influence things from the beginning, I believe you feel much more motivated."* *(interview 5)* \| We need working methods that empower staff and support shared responsibility. \| Empowering methods \| Workplace Development \| Establishing empowering working methods \| |
| --- | --- | --- | --- | --- | --- | --- | --- | --- | --- | --- | --- | --- | --- | --- | --- | --- | --- | --- | --- | --- | --- | --- | --- | --- | --- | --- | --- | --- | --- | --- | --- | --- | --- | --- | --- | --- | --- | --- | --- | --- | --- | --- | --- | --- | --- | --- | --- | --- | --- | --- | --- | --- | --- | --- | --- | --- | --- | --- | --- | --- | --- | --- | --- | --- | --- | --- | --- | --- | --- | --- | --- | --- | --- | --- | --- | --- | --- | --- | --- | --- | --- | --- | --- | --- | --- | --- | --- | --- | --- | --- | --- | --- | --- | --- | --- | --- | --- | --- |
