# Supplementary material for: Developing person-centred leadership practices: Health and social care leaders’ experiences following an educational programme
Source: PLoS One. 2026 Jul 22;21(7):e0354356. doi: 10.1371/journal.pone.0354356 (PMC13390939; doi:10.1371/journal.pone.0354356)
Supplement: Attachment — Submitted filename: Response_to reviewers (1).docx [file pone.0354356.s005.docx]

| **Editor** | **Response to comments** | **Changes made** |
| --- | --- | --- |
| *Thank you for submitting your manuscript examining participation in a person-centred leadership programme and its influence on leadership practices in health and social care. Both reviewers recognize the relevance of the topic, the clarity of the writing, and the overall strength of the manuscript.*  *While one reviewer recommends minor revision, the second raises substantive concerns that require further attention before the manuscript can be considered for publication.*  *In particular, revisions are needed to ensure alignment between the qualitative design and the claims made, especially in relation to interpretations of behavioural or cultural change. The framing of the study aim and aspects of the discussion should be adjusted to reflect the nature of the data collected.* | *Thank you for the constructive feedback and for the opportunity to revise our manuscript. We appreciate the reviewers’ thoughtful comments, which have helped us to strengthen the work.*  *In response to second Reviewers concerns, we have reformulated the study aim to ensure clearer alignment with the qualitative design. We have also revised the discussion to adjust the interpretations of our findings in relation to the methodological approach used. Although we have ongoing quantitative studies indicating significant changes in the application of personcentred practices when comparing baseline data with measurements after the programme and six months postcompletion, we fully acknowledge that such claims should not be inferred from the qualitative material presented in this manuscript. We have therefore moderated the discussion accordingly* | Abstract  p.2 line 21-23  Aim  Discussion  p. 24-25  line 548-550  line 553-554  line 570  p.26  line 612  line 614-615  line 621-622  line 636-639  line 648  line 650 |
| *In addition, clarification is required regarding compliance with the journal’s data availability policy.* | *We have reviewed the data availability section and ensured that the data are now accessible through the university’s central repository rather than via the corresponding author.* | Submission system |
| *The manuscript would also benefit from strengthened analytic transparency and a clearer discussion of reflexivity, including consideration of potential bias and the absence of negative experiences reported.* | *We have clarified the analytic process by providing a step‑by‑step description and adding a table that makes the link between raw data, coding, and theme development transparent.*  *We also acknowledge the critique regarding the absence of negative experiences and have further clarified in the results that variation was present in the data. In the discussion, we elaborate on why the findings were predominantly positive and outline possible explanations for this pattern.*  *Additionally, we have strengthened our reflexive account to ensure greater analytic rigor and transparency.* | Methods  S1 Table 1  Limitations  p.29  line 665-666  line 668-672  line 679-682  line 683-685 |
| *If applicable, we recommend that you deposit your laboratory protocols in protocols.io to enhance the reproducibility of your results. Protocols.io assigns your protocol its own identifier (DOI) so that it can be cited independently in the future.* | *Thank you for the suggestion. This recommendation is not applicable to our study, as no laboratory protocols were used.* |  |
| *Please ensure that your manuscript meets PLOS ONE's style requirements, including those for file naming. The PLOS ONE style templates can be found at*  [*https://journals.plos.org/plosone/s/file?id=wjVg/PLOSOne_formatting_sample_main_body.pdf*](https://journals.plos.org/plosone/s/file?id=wjVg/PLOSOne_formatting_sample_main_body.pdf) *and*  [*https://journals.plos.org/plosone/s/file?id=ba62/PLOSOne_formatting_sample_title_authors_affiliations.pdf*](https://track.editorialmanager.com/CL0/https:%2F%2Fjournals.plos.org%2Fplosone%2Fs%2Ffile%3Fid=ba62%2FPLOSOne_formatting_sample_title_authors_affiliations.pdf/1/010f019c58137d09-ae143208-c568-4a81-a0f8-b753bc31f757-000000/byCkrx51SyRSKmnztPopNtqihx2LfoijIwAC1_S2lzk=250) | *Thank you for noting this. We have reviewed the manuscript and ensured that it now fully complies with PLOS ONE’s style requirements, including the guidelines for file naming.* | Page 1  Submission system |
| *We note that the grant information you provided in the ‘Funding Information’ and ‘Financial Disclosure’ sections do not match.  When you resubmit, please ensure that you provide the correct grant numbers for the awards you received for your study in the ‘Funding Information’ section.* | *We have corrected the grant information, and the details in the ‘Funding Information’ and ‘Financial Disclosure’ sections now match.* | Submission system |
| *Thank you for stating the following financial disclosure: The study was financed through funding from the Gothenburg University Centre for Person-Centred Care (GPCC), Sweden.*  *URL:* [*https://www.gu.se/en/gpcc*](https://www.gu.se/en/gpcc)  *GPCC is funded by the Swedish Government’s grant for Strategic Research Areas (Care Sciences) and the University of Gothenburg, Sweden.*  *Grant numbers:*  *Eric Carlström Dnr. 2022/1512*  *Emmelie Barenfeld Dnr. 2023/837 and 2024/708.*  *The funders had no role in designing the study nor in the data collection, analysis, decision to publish or preparation of the manuscript.*  *Please state what role the funders took in the study. If the funders had no role, please state: "The funders had no role in study design, data collection and analysis, decision to publish, or preparation of the manuscript." If this statement is not correct you must amend it as needed. Please include this amended Role of Funder statement in your cover letter; we will change the online submission form on your behalf.* | *We have added the required Role of Funder statement as requested. The following wording has now been included: “The funders had no role in study design, data collection and analysis, decision to publish, or preparation of the manuscript.”* | Submission system |
| *In this instance it seems there may be acceptable restrictions in place that prevent the public sharing of your minimal data. However, in line with our goal of ensuring long-term data availability to all interested researchers, PLOS’ Data Policy states that authors cannot be the sole named individuals responsible for ensuring data access (*[*http://journals.plos.org/plosone/s/data-availability#loc-acceptable-data-sharing-methods*](http://journals.plos.org/plosone/s/data-availability#loc-acceptable-data-sharing-methods)*).  Data requests to a non-author institutional point of contact, such as a data access or ethics committee, helps guarantee long term stability and availability of data. Providing interested researchers with a durable point of contact ensures data will be accessible even if an author changes email addresses, institutions, or becomes unavailable to answer requests.  Before we proceed with your manuscript, please also provide non-author contact information (phone/email/hyperlink) for a data access committee, ethics committee, or other institutional body to which data requests may be sent. If no institutional body is available to respond to requests for your minimal data, please consider if there any institutional representatives who did not collaborate in the study, and are not listed as authors on the manuscript, who would be able to hold the data and respond to external requests for data access? If so, please provide their contact information (i.e., email address). Please also provide details on how you will ensure persistent or long-term data storage and availability.* | *We have updated our* Data Availability Statement  *A DOI to a description of the dataset will be provided upon acceptance* | Submission system Data Availability Statement |
| *Please include captions for your Supporting Information files at the end of your manuscript, and update any in-text citations to match accordingly. Please see our Supporting Information guidelines for more information:* [*http://journals.plos.org/plosone/s/supporting-information*](http://journals.plos.org/plosone/s/supporting-information) | *Thank you for the instruction. We have made sure that captions for all Supporting Information files are included at the end of the manuscript and updated the intext citations accordingly.* | Page 31 |
| *If the reviewer comments include a recommendation to cite specific previously published works, please review and evaluate these publications to determine whether they are relevant and should be cited. There is no requirement to cite these works unless the editor has indicated otherwise.* | *This point is not applicable, as no reviewer requested that we cite specific previously published works.* | n.a. |
| ***Reviewer 1*** | **Response to comments** | **Changes made** |
| *Well done in continuing the work in PCC leadership evaluation. Clear paper with logical flow to findings. Consider adding more evidence from participant quotes to support your findings. Nice use of international application and evidence.* | *Thank you for the positive and encouraging feedback on the clarity and contribution of our work. We considered adding more participant quotes; however, the results section is already lengthy. Instead, we have strengthened the linkage between raw data and findings by referring to an Appendix in the methods section including a table overviewing the analytical process. In this table we have added additional quotes to transparently show how raw data map to codes and themes.* | S2 Appendix |
| ***Reviewer 2*** | **Response to comments** | **Changes made** |
| *This manuscript sets out to explore how participation in an educational programme on* *person-centred leadership in Sweden influenced the leadership practices of health and social care leaders trough individual interviews with 13 participants in the programme. However, given the study design it is questionable if the authors have captured the influences of leadership practices. I would rather say that the study is about experiences of participating in the programme and the self perceived changes of leadership practices which have been studied. Therefore, I suggest changing the aim slightly.* | *Thank you for this helpful comment. We agree that the original aim did not fully reflect the nature of the data and what could be meaningfully captured through the study design. In line with your suggestion, we have revised the aim to more accurately describe the focus of the study. The revised aim now reads: “to explore experiences of selfperceived‑perceived changes in leadership practices following participation in a person‑centred leadership programme”.* | Aim is modified, trough out the manuscript  Abstract  p.2 line 22-24  Aim  Discussion  p. 24-25  line 549-551 |
| *The study addresses a topical issue, it is well-written and well-structured and easy to read and follow.* | *We sincerely appreciate your positive assessment of the manuscript. Your acknowledgement of the topic’s relevance, the clarity of the writing, and the overall structure is encouraging and greatly valued.* | n.a. |
| *The abstract and key-words describe the study in a clear manner.* |  |  |
| *The background sets the scene and up-to-date international references are used.* |  |  |
| *The methods are sound and it is easy to follow how sampling, data collection and analysis were conducted. The analysis section would be even more clear if a table of figure was inserted with the steps in the coding, from quote to theme.* | *Thank you for this constructive suggestion. We have added a table A in S2 Appendix to clarify the analytic process and to illustrate the steps from raw quotes to codes sub-categories, categories and the overarching theme, as recommended.* | p.11  line 222-223,  S2 Appendix |
| *In the discussion section, I think the authors make some claims which are not valid, for instance row 610- 615: "Our findings describe that such programmes can successfully promote behavioural change by encouraging participants to prioritise understanding both employee and patient needs and to value curious exploration over assumptions. Participants developed their maturity and leadership capabilities, focusing on refining their leadership styles and deepening their connections with employees through practising work in partnership during the programme". This has not been studied, as we know nothing about actual change in leadership behaviour, we merely know how leaders experienced that the programme has influenced them.* | Thank you for this important observation. We agree that the original phrasing implied conclusions that extended beyond what could be supported by the qualitative data. We have addressed this by revising the study aim and by adjusting our phrasing in the results and the discussion to avoid claims about actual behavioural change. The revised discussion now focuses on participants’ self‑perceived influences from the programme, in line with what was studied. | Aim  Results  Line  237,241, 291, 330, 346  Discussion  p.27  line 613  line 615-616  line 622-623  line 637-640  Conclusion |
| *Furthermore, the same goes for statements on row 620-621 "Finally, viewing the transition toward PCC through the lens of workforce development reveals how leadership programmes can act as catalysts for cultural and behavioural change.". To make such claims, the actual leadership and the perspectives on employee and organisation need to be investigated. Please make more modest claims that align with the study design.* | *As noted earlier, we have revised the discussion to ensure that claims align with the qualitative design and focus on participants’ self‑perceived influences. While ongoing quantitative studies in the wider project indicate that such changes may occur, we acknowledge that referring to these results led to overly strong conclusions in this manuscript. The language has therefore been moderated accordingly.* | Discussion  line 620-623  line 637-640  Conclusion |
| *Finally, I found if puzzling that there seemed to be no negative experiences of programme-participation. Usually, there are a number of mixed experiences of educational efforts, and one cannot but wonder if the overly positive experience are biases as two of the authors arranged the course? I would like to see some further elaboration in this matter.* | *Thank you for raising this important point. We acknowledge the concern regarding the absence of explicitly negative experiences in the findings. In line with our earlier response to the editor, we have clarified both in the results and discussion that variation was present in participants’ accounts, while also elaborating on why predominantly positive experiences may have been reported. We have additionally strengthened our reflexive considerations, including reflecting on the potential influence of the authors’ involvement in the programme.* | Abstract  Line  34  Results  Line  241  Discussion  Line  554-555  564-567  615-616  620  Limitations  Line  666-667  669-671, 673  684-686 |
|  | *We have also nuanced the language in the conclusions inline with the changes above* | Conclusion |
