# Supplementary material for: Developing person-centred leadership practices: Health and social care leaders’ experiences following an educational programme
Source: PLoS One. 2026 Jul 22;21(7):e0354356. doi: 10.1371/journal.pone.0354356 (PMC13390939; doi:10.1371/journal.pone.0354356)
Supplement: Attachment — Submitted filename: Response to reviewers.docx [file pone.0354356.s006.docx]

| Editor Comments |  |
| --- | --- |
| Thank you for your careful revision of the manuscript and for your patience. The revised submission has successfully addressed the substantive concerns raised during the previous (initial) review round, and the study will likely make a valuable contribution to the literature on person centered leadership development in health and social care settings.  Following this review cycle, I am requesting a minor revision before a final publication decision is made. The requested revisions are intended primarily to strengthen methodological transparency and reporting clarity rather than to require additional analyses or substantial restructuring of the manuscript.  Specifically, please consider: | Dear Editor and reviewers,  Thank you for your careful review and constructive feedback. We appreciate the opportunity to revise the manuscript and further strengthen its methodological transparency and clarity. We have addressed the points raised, as detailed in the responses to provided comments. |
| Expanding the description of the educational programme in the Methods section to provide readers with sufficient context without requiring consultation of another publication. | In the methods section information on the Swedish health and social care context and the programme have been added. We have also restructured descriptions of the programme to improve readability.  Page: 5-7  Heading: Study setting  Line: 114-123, 125, 128-140, 151-154, 160-169  New references added: 26, 27, 28 |
| Clarifying the sampling strategy, including the relationship between the total eligible population and the final study sample, the rationale for participant selection, and the characteristics used to ensure variation within the sample. | We have extended the Methods section in line with the comment to clarify the sampling strategy and variation in participant characteristics.  Page: 8-9 Method, Participants  Line: 185-186, 190-191,  Moreover, we have commented on the representativeness of the selected participants relative to the broader programme cohort.  Line: 188-191 |
| Providing a more detailed description of participant characteristics (i.e., leadership roles, experience, and other relevant demographic information where available and appropriate). | The requested participant characteristics (e.g., leadership roles, experience, and demographic information such as age, gender, work setting) are already presented in Table 1. To further improve clarity, we have explicitly highlighted this information in the main text.  Page: 9 Method, Participants  Line: 197-198 Table1 |
| Moving or incorporating discussion of adherence to established qualitative reporting criteria into the Methods section, where it may be more appropriately presented as a methodological strength. | The information on how COREQ was applied has been moved to the Methods section, along with a clarification of how it contributes to trustworthiness.  Page: 13 Method, Establishing trustworthiness Line: 280-282  New references added: 38 |
| Strengthening the discussion of trustworthiness, methodological rigor, reflexivity, and transferability, including consideration of how participant characteristics and contextual factors may have influenced the findings and their applicability to other settings. | We have added a section of Establishing trustworthiness in the methods section, where we have included new information and reorganized parts of the methods and methods discussion to bring together all aspects related to establishing trustworthiness in one place.  Page: 12-13 Method, Establishing trustworthiness  Moreover, we have further developed the discussion in line with the comment as well as the methodological limitation section.  Page: 30 Discussion Line: 701-704  Page: 31-32 Limitations Line: 722-735, 738-745, 747-748 |
| These revisions are achievable without substantial changes to the study design, analysis, or conclusions. Addressing these points will further strengthen the manuscript and enhance its contribution to an international readership. | We appreciate this observation and have revised the manuscript to address these points, further strengthening its clarity and contribution. |
| Reviewers' comments: |  |
| **Reviewer #2:** |  |
| The authors have attended to my comments in a constructive way, and the manuscript is considerably improved. I have no further comments or queries. | Thank you |
| **Reviewer #3:** |  |
| Overall, the paper addresses an interesting and relevant topic by qualitatively exploring health and social care leaders’ experiences of changes in leadership practices following their participation in an educational programme on person-centred leadership. | We appreciate this positive assessment of the study and its relevance to the field. |
| The study highlights several important aspects. Notably, it underscores the importance of integrating practical assignments into workplace settings, addressing everyday challenges, and involving leaders, employees, patients, and their relatives at an early stage. Such an approach supports the continuous development of leadership practices and facilitates the sustainable transformation of health and social care towards person-centred care. |  |
| The background is both relevant and engaging and a nice introduction to PCC. In the last sentence in page 7, line 157-158 you mention that further details on the programme are provided by Lood et al. (reference 15), consider adding some more details of the programme here to introduce the reader to shed light on a few details. And you will have a clearer connection to the heading participants and what they perceived after the programme ended. | Thank you for this constructive feedback. In line with the editor’s comment, we have expanded the description of the programme in the Methods section to provide additional context and strengthen the connection to the participants’ experiences following the programme.  Page: 5-8 Method: Study setting  Line: 114-123,  Method: The educational programme  Line: 129-140, 151-154. 157-158, 160-169  Moreover, we have added a sentence in the Introduction that directs the reader to the description of the educational programme in the Methods section.  Page: 5, Introduction  Line: 109-110 |
| On page 7, line 161 (Participants), the sentence states that a selection of participants was used. Consider adding some more information about the sampling, was it a purposive sampling with a strategic strategy where you have criteria’s? How was the total population in relation to your selected population? | We have clarified that the sampling strategy was purposeful and which criteria we applied in the methods section.  Page: 8 Method: Participants  Line: 185-186, 188-91 |
| Specifically, which characteristics were considered important to ensure variation? I therefore recommend clarify this section to describe the total sample characteristics more explicitly. Was the total sample n=13? As it stands, the sampling approach seems reflective of maximum variation and indicative of purposive sampling based on the target population. But when you write that four participants had primary responsibility for the implementation and compliance of PCC, while nine were actively involved in leading, were the total sample or do you have more persons available for example responsible for the implementations. | Please see the previous response.  Yes, the total sample consisted of 13 participants. We have clarified in the text what is meant by having primary responsibility for the implementation of PCC. While several of the leaders had responsibility and mandate within their own units, they did not hold overall responsibility at the organizational level. This distribution is consistent with the proportion of participants included in the interviews relative to participants in the programme.  Page: 9 Method: Participants  Line: 197-198, 200-202 |
| It is very nice that you have participants in your sample from different regions across Sweden and worked in various health and social care sectors. | We appreciate this positive comment and the recognition of the sample’s diversity. |
| Consider adding information in the Methods section indicating that you followed established criteria for reporting qualitative research. This is currently mentioned only in the final sentence of the Limitations section (p. 30). However, from my perspective, this is more appropriately presented as a methodological strength rather than a limitation. | This has been presented as a methodological strength of the reporting under the subsection *Establishing trustworthiness* in the Methods section.  Page: 13 Method: Establishing trustworthiness  Line: 280-282 |
| The Discussion section, as well as the sections addressing study limitations and strengths, could more explicitly engage with issues of trustworthiness, including reflexivity and methodological rigor. A more thorough reflection on these aspects would strengthen the overall transparency and credibility of the study. | In line with your previous comment, we have chosen to consolidate the actions we have taken under the heading *“Establishing trustworthiness”* in the Methods section. To enable a more focused and in-depth discussion, we have moved relevant content from the limitations discussion to this section. This restructuring has allowed us to better address your and the editors’ feedback regarding aspects that require further elaboration and critical discussion under the heading Limitation.  Page: 12-13 Method: Establishing trustworthiness  New references added: 38 Page: 32 Limitations Line: 749-755 |
| In addition, it would be valuable to provide a clearer and more detailed description of the sample characteristics, such as sex/gender, age, professional roles, and years of experience in leadership positions.  Furthermore, reflecting on how these factors may have influenced the findings would add important analytical depth. Such considerations would also enhance the discussion of the transferability of the findings to other contexts. | The requested information on participants’ characteristics (including sex/gender, age, leadership roles, and years of experience) was collected and is presented in Table 1. To improve clarity, we have strengthened the linkage to Table 1 in the manuscript text by explicitly referring to and briefly summarising the sample characteristics.  Page: 9 Line: 197-198 Method Participants  Reflections on how participant characteristics may have influenced the findings are included in the limitations section. There, we also discuss how this may affect the transferability of the results.  Page: 31-32 Limitations Line: 722-745  New references added: 57-58 |
| Overall, a more comprehensive description and critical discussion of these elements would improve the clarity and coherence of the argument and strengthen reflections on both the study context and the potential applicability of the findings to a broader international audience. | We have expanded the description of the Swedish health care system to clarify the study context and strengthen the international relevance of our findings. The role of the study context for transferability is also addressed.  Page: 5-6  Method, Study setting  Line: 114-123, 129-139  New references added: 26-28  Page: 30-31 Discussion Line: 701-704 Limitations: 722-745, 747-748 |
| Despite these suggestions for further development, the study represents a valuable contribution to the advancement of knowledge within this field of developing person-centred leadership practice. | Thank you for valuable constructive feedback and for highlighting the strengths of the study. |
|  |  |
