# Supplementary material for: Developing person-centred leadership practices: Health and social care leaders’ experiences following an educational programme
Source: PLoS One. 2026 Jul 22;21(7):e0354356. doi: 10.1371/journal.pone.0354356 (PMC13390939; doi:10.1371/journal.pone.0354356)
Supplement: Peer review history

# Peer review: Decision Letter 0

**DOI:** 10.1371/journal.pone.0354356.r001

**Authors:** Philip Cola

13 Feb 2026

PONE-D-25-37185

Developing person-centred leadership practices: The influence of an educational programme for health and social care leaders in the transition towards person-centred care

PLOS One

Dear Dr. Barenfeld,

Thank you for submitting your manuscript to PLOS ONE. After careful consideration, we feel that it has merit but does not fully meet PLOS ONE’s publication criteria as it currently stands. Therefore, we invite you to submit a revised version of the manuscript that addresses the points raised during the review process.

Thank you for submitting your manuscript examining participation in a person-centred leadership programme and its influence on leadership practices in health and social care. Both reviewers recognize the relevance of the topic, the clarity of the writing, and the overall strength of the manuscript.

While one reviewer recommends minor revision, the second raises substantive concerns that require further attention before the manuscript can be considered for publication.

In particular, revisions are needed to ensure alignment between the qualitative design and the claims made, especially in relation to interpretations of behavioural or cultural change. The framing of the study aim and aspects of the discussion should be adjusted to reflect the nature of the data collected.

In addition, clarification is required regarding compliance with the journal’s data availability policy. The manuscript would also benefit from strengthened analytic transparency and a clearer discussion of reflexivity, including consideration of potential bias and the absence of negative experiences reported.

We believe these concerns are addressable through careful revision and invite you to submit a revised manuscript along with a detailed response to the reviewers’ comments.

We look forward to receiving your revision.

Please submit your revised manuscript by Mar 30 2026 11:59PM. If you will need more time than this to complete your revisions, please reply to this message or contact the journal office at plosone@plos.org. When you’re ready to submit your revision, log on to https://www.editorialmanager.com/pone/ and select the ‘Submissions Needing Revision’ folder to locate your manuscript file.

Please include the following items when submitting your revised manuscript:

- A letter that responds to each point raised by the academic editor and reviewer(s). You should upload this letter as a separate file labeled 'Response to Reviewers'.
- A marked-up copy of your manuscript that highlights changes made to the original version. You should upload this as a separate file labeled 'Revised Manuscript with Track Changes'.
- An unmarked version of your revised paper without tracked changes. You should upload this as a separate file labeled 'Manuscript'.

If you would like to make changes to your financial disclosure, please include your updated statement in your cover letter. Guidelines for resubmitting your figure files are available below the reviewer comments at the end of this letter.

If applicable, we recommend that you deposit your laboratory protocols in protocols.io to enhance the reproducibility of your results. Protocols.io assigns your protocol its own identifier (DOI) so that it can be cited independently in the future. For instructions see: https://journals.plos.org/plosone/s/submission-guidelines#loc-laboratory-protocols. Additionally, PLOS ONE offers an option for publishing peer-reviewed Lab Protocol articles, which describe protocols hosted on protocols.io. Read more information on sharing protocols at https://plos.org/protocols?utm_medium=editorial-email&utm_source=authorletters&utm_campaign=protocols.

We look forward to receiving your revised manuscript.

Kind regards,

Philip A. Cola, Ph.D.

Academic Editor

PLOS One

Journal Requirements:

When submitting your revision, we need you to address these additional requirements.

1. Please ensure that your manuscript meets PLOS ONE's style requirements, including those for file naming. The PLOS ONE style templates can be found at

https://journals.plos.org/plosone/s/file?id=wjVg/PLOSOne_formatting_sample_main_body.pdf and

https://journals.plos.org/plosone/s/file?id=ba62/PLOSOne_formatting_sample_title_authors_affiliations.pdf

2. We note that the grant information you provided in the ‘Funding Information’ and ‘Financial Disclosure’ sections do not match.

When you resubmit, please ensure that you provide the correct grant numbers for the awards you received for your study in the ‘Funding Information’ section.

3. Thank you for stating the following financial disclosure:

The study was financed through funding from the Gothenburg University Centre for Person-Centred Care (GPCC), Sweden.

URL: https://www.gu.se/en/gpcc

GPCC is funded by the Swedish Government’s grant for Strategic Research Areas (Care Sciences) and the University of Gothenburg, Sweden.

Grant numbers:

Eric Carlström Dnr. 2022/1512

Emmelie Barenfeld Dnr. 2023/837 and 2024/708.

The funders had no role in designing the study nor in the data collection, analysis, decision to publish or preparation of the manuscript.

Please state what role the funders took in the study. If the funders had no role, please state: "The funders had no role in study design, data collection and analysis, decision to publish, or preparation of the manuscript."

If this statement is not correct you must amend it as needed.

Please include this amended Role of Funder statement in your cover letter; we will change the online submission form on your behalf.

4. In this instance it seems there may be acceptable restrictions in place that prevent the public sharing of your minimal data. However, in line with our goal of ensuring long-term data availability to all interested researchers, PLOS’ Data Policy states that authors cannot be the sole named individuals responsible for ensuring data access (http://journals.plos.org/plosone/s/data-availability#loc-acceptable-data-sharing-methods).

Data requests to a non-author institutional point of contact, such as a data access or ethics committee, helps guarantee long term stability and availability of data. Providing interested researchers with a durable point of contact ensures data will be accessible even if an author changes email addresses, institutions, or becomes unavailable to answer requests.

Before we proceed with your manuscript, please also provide non-author contact information (phone/email/hyperlink) for a data access committee, ethics committee, or other institutional body to which data requests may be sent. If no institutional body is available to respond to requests for your minimal data, please consider if there any institutional representatives who did not collaborate in the study, and are not listed as authors on the manuscript, who would be able to hold the data and respond to external requests for data access? If so, please provide their contact information (i.e., email address). Please also provide details on how you will ensure persistent or long-term data storage and availability.

5. Please include captions for your Supporting Information files at the end of your manuscript, and update any in-text citations to match accordingly. Please see our Supporting Information guidelines for more information: http://journals.plos.org/plosone/s/supporting-information.

6. If the reviewer comments include a recommendation to cite specific previously published works, please review and evaluate these publications to determine whether they are relevant and should be cited. There is no requirement to cite these works unless the editor has indicated otherwise.

Additional Editor Comments:

Thank you for submitting your manuscript examining participation in a person-centred leadership programme and its influence on leadership practices in health and social care. Both reviewers recognize the relevance of the topic, the clarity of the writing, and the overall strength of the manuscript.

After consideration of the reviews, the decision is Major Revision.

While one reviewer recommends minor revision, the second raises substantive concerns that require further attention before the manuscript can be considered for publication. In particular, revisions are needed to ensure alignment between the qualitative design and the claims made, especially in relation to interpretations of behavioural or cultural change. The framing of the study aim and aspects of the discussion should be adjusted to reflect the nature of the data collected.

In addition, clarification is required regarding compliance with the journal’s data availability policy. The manuscript would also benefit from strengthened analytic transparency and a clearer discussion of potential positionality, including consideration of potential bias and the absence of negative experiences reported.

We believe these concerns are addressable through careful revision and invite you to submit a revised manuscript along with a detailed response to the reviewers’ comments.

We look forward to receiving your revision.

[Note: HTML markup is below. Please do not edit.]

Reviewers' comments:

Reviewer's Responses to Questions

**Comments to the Author**

1. Is the manuscript technically sound, and do the data support the conclusions?

The manuscript must describe a technically sound piece of scientific research with data that supports the conclusions. Experiments must have been conducted rigorously, with appropriate controls, replication, and sample sizes. The conclusions must be drawn appropriately based on the data presented.

Reviewer #1: Yes

Reviewer #2: Partly

**********

2. Has the statistical analysis been performed appropriately and rigorously?

Reviewer #1: N/A

Reviewer #2: N/A

**********

3. Have the authors made all data underlying the findings in their manuscript fully available?

The PLOS Data policy requires authors to make all data underlying the findings described in their manuscript fully available without restriction, with rare exception (please refer to the Data Availability Statement in the manuscript PDF file). The data should be provided as part of the manuscript or its supporting information, or deposited to a public repository. For example, in addition to summary statistics, the data points behind means, medians and variance measures should be available. If there are restrictions on publicly sharing data—e.g. participant privacy or use of data from a third party—those must be specified.

Reviewer #1: Yes

Reviewer #2: No

**********

4. Is the manuscript presented in an intelligible fashion and written in standard English?

PLOS ONE does not copyedit accepted manuscripts, so the language in submitted articles must be clear, correct, and unambiguous. Any typographical or grammatical errors should be corrected at revision, so please note any specific errors here.

Reviewer #1: Yes

Reviewer #2: Yes

**********

5. Review Comments to the Author

Please use the space provided to explain your answers to the questions above. You may also include additional comments for the author, including concerns about dual publication, research ethics, or publication ethics. (Please upload your review as an attachment if it exceeds 20,000 characters)

Reviewer #1: Well done in continuing the work in PCC leadership evaluation. Clear paper with logical flow to findings. Consider adding more evidence from participant quotes to support your findings. Nice use of international application and evidence.

Reviewer #2: This manuscript sets out to explore how participation in an educational programme on

person-centred leadership in Sweden influenced the leadership practices of health and social care leaders trough individual interviews with 13 participants in the programme. However, given the study design it is questionable if the authors have captured the influences of leadership practices. I would rather say that the study is about experiences of participating in the programme and the self perceived changes of leadership practices which have been studied. Therefore, I suggest changing the aim slightly.

The study addresses a topical issue, it is well-written and well-structured and easy to read and follow.

The abstract and key-words describe the study in a clear manner.

The background sets the scene and up-to-date international references are used.

The methods are sound and it is easy to follow how sampling, data collection and analysis were conducted. The analysis section would be even more clear if a table of figure was inserted with the steps in the coding, from quote to theme.

In the discussion section, I think the authors make some claims which are not valid, for instance row 610- 615: "Our findings describe that such programmes can successfully promote behavioural change by encouraging participants to prioritise understanding both employee and patient needs and to value curious exploration over assumptions. Participants developed their maturity and leadership capabilities, focusing on refining their leadership

styles and deepening their connections with employees through practising work in partnership

during the programme". This has not been studied, as we know nothing about actual change in leadership behaviour, we merely know how leaders experienced that the programme has influenced them. Furthermore, the same goes for statements on on row 620-621 "Finally, viewing the transition toward PCC through the lens of workforce development reveals how leadership programmes can act as catalysts for cultural and behavioural change.". To make such claims, the actual leadership and the perspectives on employee and organisation need to be investigated. Please make more modest claims that align with the study design.

Finally, I found if puzzling that there seemed to be no negative experiences of programme-participation. Usually, there are a number of mixed experiences of educational efforts, and one cannot but wonder if the overly positive experience are biases as two of the authors arranged the course? I would like to see some further elaboration in this matter.

**********

6. PLOS authors have the option to publish the peer review history of their article (what does this mean?). If published, this will include your full peer review and any attached files.

If you choose “no”, your identity will remain anonymous but your review may still be made public.

**Do you want your identity to be public for this peer review?**  For information about this choice, including consent withdrawal, please see our Privacy Policy.

Reviewer #1: No

Reviewer #2: No

**********

[NOTE: If reviewer comments were submitted as an attachment file, they will be attached to this email and accessible via the submission site. Please log into your account, locate the manuscript record, and check for the action link "View Attachments". If this link does not appear, there are no attachment files.]

To ensure your figures meet our technical requirements, please review our figure guidelines: https://journals.plos.org/plosone/s/figures

You may also use PLOS’s free figure tool, NAAS, to help you prepare publication quality figures: https://journals.plos.org/plosone/s/figures#loc-tools-for-figure-preparation.

NAAS will assess whether your figures meet our technical requirements by comparing each figure against our figure specifications.

# Author comment: Author response to Decision Letter 1

**DOI:** 10.1371/journal.pone.0354356.r002

19 Mar 2026

Dear editor and reviewers,

Thank you for the constructive feedback and for the opportunity to revise our manuscript. We appreciate the reviewers’ thoughtful comments, which have helped us to strengthen the work. Please see attached document Response to Reviewers for our replies. Best Regards, Emmelie Barenfeld on behalf of all co-authors

Attachment Submitted filename: Response_to reviewers (1).docx

# Peer review: Decision Letter 1

**DOI:** 10.1371/journal.pone.0354356.r003

**Authors:** Philip Cola

3 Jun 2026

PONE-D-25-37185R1

Developing person-centred leadership practices: Health and social care leaders’ experiences following an educational programme

PLOS One

Dear Dr. Barenfeld,

Thank you for submitting your manuscript to PLOS ONE. After careful consideration, we feel that it has merit but does not fully meet PLOS ONE’s publication criteria as it currently stands. Therefore, we invite you to submit a revised version of the manuscript that addresses the points raised during the review process.

After consideration of the revised manuscript and additional reviewer feedback, we are requesting Minor Revisions.

The manuscript has satisfactorily addressed the major concerns raised during the previous (initial) review round and represents a valuable contribution to the literature on person centered leadership in health and social care. The remaining issues relate primarily to methodological transparency, reporting clarity, and discussion of trustworthiness and transferability. I encourage the authors to address these final points, after which I anticipate being able to make a final decision on the manuscript.

Please submit your revised manuscript by Jul 18 2026 11:59PM. If you will need more time than this to complete your revisions, please reply to this message or contact the journal office at plosone@plos.org. When you're ready to submit your revision, log on to https://www.editorialmanager.com/pone/ and select the 'Submissions Needing Revision' folder to locate your manuscript file.

Please include the following items when submitting your revised manuscript:

- A letter that responds to each point raised by the academic editor and reviewer(s). You should upload this letter as a separate file labeled 'Response to Reviewers'.
- A marked-up copy of your manuscript that highlights changes made to the original version. You should upload this as a separate file labeled 'Revised Manuscript with Track Changes'.
- An unmarked version of your revised paper without tracked changes. You should upload this as a separate file labeled 'Manuscript'.

If you would like to make changes to your financial disclosure, please include your updated statement in your cover letter. Guidelines for resubmitting your figure files are available below the reviewer comments at the end of this letter.

If applicable, we recommend that you deposit your laboratory protocols in protocols.io to enhance the reproducibility of your results. Protocols.io assigns your protocol its own identifier (DOI) so that it can be cited independently in the future. For instructions see: https://journals.plos.org/plosone/s/submission-guidelines#loc-laboratory-protocols. Additionally, PLOS ONE offers an option for publishing peer-reviewed Lab Protocol articles, which describe protocols hosted on protocols.io. Read more information on sharing protocols at https://plos.org/protocols?utm_medium=editorial-email&utm_source=authorletters&utm_campaign=protocols.

As the corresponding author, your ORCID iD is verified in the submission system and will appear in the published article. PLOS supports the use of ORCID, and we encourage all coauthors to register for an ORCID iD and use it as well. Please encourage your coauthors to verify their ORCID iD within the submission system before final acceptance, as unverified ORCID iDs will not appear in the published article. *Only*  the individual author can complete the verification step; PLOS staff *cannot*  verify ORCID iDs on behalf of authors.

We look forward to receiving your revised manuscript.

Kind regards,

Philip A. Cola, Ph.D.

Academic Editor

PLOS One

Journal Requirements:

If the reviewer comments include a recommendation to cite specific previously published works, please review and evaluate these publications to determine whether they are relevant and should be cited. There is no requirement to cite these works unless the editor has indicated otherwise.

Please review your reference list to ensure that it is complete and correct. If you have cited papers that have been retracted, please include the rationale for doing so in the manuscript text, or remove these references and replace them with relevant current references. Any changes to the reference list should be mentioned in the rebuttal letter that accompanies your revised manuscript. If you need to cite a retracted article, indicate the article’s retracted status in the References list and also include a citation and full reference for the retraction notice.

Additional Editor Comments:

Thank you for your careful revision of the manuscript and for your patience. The revised submission has successfully addressed the substantive concerns raised during the previous (initial) review round, and the study will likely make a valuable contribution to the literature on person centered leadership development in health and social care settings.

Following this review cycle, I am requesting a minor revision before a final publication decision is made. The requested revisions are intended primarily to strengthen methodological transparency and reporting clarity rather than to require additional analyses or substantial restructuring of the manuscript.

Specifically, please consider:

Expanding the description of the educational programme in the Methods section to provide readers with sufficient context without requiring consultation of another publication.

Clarifying the sampling strategy, including the relationship between the total eligible population and the final study sample, the rationale for participant selection, and the characteristics used to ensure variation within the sample.

Providing a more detailed description of participant characteristics (i.e., leadership roles, experience, and other relevant demographic information where available and appropriate).

Moving or incorporating discussion of adherence to established qualitative reporting criteria into the Methods section, where it may be more appropriately presented as a methodological strength.

Strengthening the discussion of trustworthiness, methodological rigor, reflexivity, and transferability, including consideration of how participant characteristics and contextual factors may have influenced the findings and their applicability to other settings.

These revisions are achievable without substantial changes to the study design, analysis, or conclusions. Addressing these points will further strengthen the manuscript and enhance its contribution to an international readership.

[Note: HTML markup is below. Please do not edit.]

Reviewers' comments:

Reviewer's Responses to Questions

**Comments to the Author**

1. If the authors have adequately addressed your comments raised in a previous round of review and you feel that this manuscript is now acceptable for publication, you may indicate that here to bypass the “Comments to the Author” section, enter your conflict of interest statement in the “Confidential to Editor” section, and submit your "Accept" recommendation.

Reviewer #2: All comments have been addressed

Reviewer #3: All comments have been addressed

**********

2. Is the manuscript technically sound, and do the data support the conclusions?

The manuscript must describe a technically sound piece of scientific research with data that supports the conclusions. Experiments must have been conducted rigorously, with appropriate controls, replication, and sample sizes. The conclusions must be drawn appropriately based on the data presented.

Reviewer #2: Yes

Reviewer #3: Partly

**********

3. Has the statistical analysis been performed appropriately and rigorously?

Reviewer #2: N/A

Reviewer #3: N/A

**********

4. Have the authors made all data underlying the findings in their manuscript fully available?

The PLOS Data policy requires authors to make all data underlying the findings described in their manuscript fully available without restriction, with rare exception (please refer to the Data Availability Statement in the manuscript PDF file). The data should be provided as part of the manuscript or its supporting information, or deposited to a public repository. For example, in addition to summary statistics, the data points behind means, medians and variance measures should be available. If there are restrictions on publicly sharing data—e.g. participant privacy or use of data from a third party—those must be specified.

Reviewer #2: Yes

Reviewer #3: Yes

**********

5. Is the manuscript presented in an intelligible fashion and written in standard English?

PLOS ONE does not copyedit accepted manuscripts, so the language in submitted articles must be clear, correct, and unambiguous. Any typographical or grammatical errors should be corrected at revision, so please note any specific errors here.

Reviewer #2: Yes

Reviewer #3: Yes

**********

6. Review Comments to the Author

Please use the space provided to explain your answers to the questions above. You may also include additional comments for the author, including concerns about dual publication, research ethics, or publication ethics. (Please upload your review as an attachment if it exceeds 20,000 characters)

Reviewer #2: The authors have attended to my comments in a constructive way, and the manuscript is considerably improved.

I have no further comments or queries.

Reviewer #3: Overall, the paper addresses an interesting and relevant topic by qualitatively exploring health and social care leaders’ experiences of changes in leadership practices following their participation in an educational programme on person-centred leadership.

The study highlights several important aspects. Notably, it underscores the importance of integrating practical assignments into workplace settings, addressing everyday challenges, and involving leaders, employees, patients, and their relatives at an early stage. Such an approach supports the continuous development of leadership practices and facilitates the sustainable transformation of health and social care towards person-centred care.

The background is both relevant and engaging and a nice introduction to PCC. In the last sentence in page 7, line 157-158 you mention that further details on the programme are provided by Lood et al. (reference 15), consider adding some more details of the programme here to introduce the reader to shed light on a few details. And you will have a clearer connection to the heading participants and what they perceived after the programme ended.

On page 7, line 161 (Participants), the sentence states that a selection of participants was used. Consider adding some more information about the sampling, was it a purposive sampling with a strategic strategy where you have criteria’s? How was the total population in relation to your selected population?

Specifically, which characteristics were considered important to ensure variation? I therefore recommend clarify this section to describe the total sample characteristics more explicitly. Was the total sample n=13? As it stands, the sampling approach seems reflective of maximum variation and indicative of purposive sampling based on the target population. But when you write that four participants had primary responsibility for the implementation and compliance of PCC, while nine were actively involved in leading, were the total sample or do you have more persons available for example responsible for the implementations. It is very nice that you have participants in your sample from different regions across Sweden and worked in various health and social care sectors.

Consider adding information in the Methods section indicating that you followed established criteria for reporting qualitative research. This is currently mentioned only in the final sentence of the Limitations section (p. 30). However, from my perspective, this is more appropriately presented as a methodological strength rather than a limitation.

The Discussion section, as well as the sections addressing study limitations and strengths, could more explicitly engage with issues of trustworthiness, including reflexivity and methodological rigor. A more thorough reflection on these aspects would strengthen the overall transparency and credibility of the study.

In addition, it would be valuable to provide a clearer and more detailed description of the sample characteristics, such as sex/gender, age, professional roles, and years of experience in leadership positions. Furthermore, reflecting on how these factors may have influenced the findings would add important analytical depth. Such considerations would also enhance the discussion of the transferability of the findings to other contexts.

Overall, a more comprehensive description and critical discussion of these elements would improve the clarity and coherence of the argument and strengthen reflections on both the study context and the potential applicability of the findings to a broader international audience.

Despite these suggestions for further development, the study represents a valuable contribution to the advancement of knowledge within this field of developing person-centred leadership practice.

**********

7. PLOS authors have the option to publish the peer review history of their article (what does this mean?). If published, this will include your full peer review and any attached files.

If you choose “no”, your identity will remain anonymous but your review may still be made public.

**Do you want your identity to be public for this peer review?**  For information about this choice, including consent withdrawal, please see our Privacy Policy.

Reviewer #2: No

Reviewer #3: No

**********

[NOTE: If reviewer comments were submitted as an attachment file, they will be attached to this email and accessible via the submission site. Please log into your account, locate the manuscript record, and check for the action link "View Attachments". If this link does not appear, there are no attachment files.]

To ensure your figures meet our technical requirements, please review our figure guidelines: https://journals.plos.org/plosone/s/figures

You may also use PLOS’s free figure tool, NAAS, to help you prepare publication quality figures: https://journals.plos.org/plosone/s/figures#loc-tools-for-figure-preparation.

NAAS will assess whether your figures meet our technical requirements by comparing each figure against our figure specifications.

# Author comment: Author response to Decision Letter 2

**DOI:** 10.1371/journal.pone.0354356.r004

24 Jun 2026

Dear Editor and reviewers,

Thank you for your careful review and constructive feedback. We appreciate the opportunity to revise the manuscript and further strengthen its methodological transparency and clarity. We have addressed the points raised, as detailed in the responses to provided comments. Please see the attached document Response to reviewers for details.

Best Regards,

Emmelie Barenfeld on behalf of all co-authors

Attachment Submitted filename: Response to reviewers.docx

# Editor report: Decision Letter 2

**DOI:** 10.1371/journal.pone.0354356.r005

**Authors:** Philip Cola

8 Jul 2026

Developing person-centred leadership practices: Health and social care leaders’ experiences following an educational programme

PONE-D-25-37185R2

Dear Dr. Barenfeld,

We’re pleased to inform you that your manuscript has been judged scientifically suitable for publication and will be formally accepted for publication once it meets all outstanding technical requirements.

Within one week, you’ll receive an e-mail detailing the required amendments. When these have been addressed, you’ll receive a formal acceptance letter and your manuscript will be scheduled for publication.

An invoice will be generated when your article is formally accepted. Please note, if your institution has a publishing partnership with PLOS and your article meets the relevant criteria, all or part of your publication costs will be covered. Please make sure your user information is up-to-date by logging into Editorial Manager at Editorial Manager® and clicking the ‘Update My Information' link at the top of the page. For questions related to billing, please contact billing support.

If your institution or institutions have a press office, please notify them about your upcoming paper to help maximize its impact. If they’ll be preparing press materials, please inform our press team as soon as possible -- no later than 48 hours after receiving the formal acceptance. Your manuscript will remain under strict press embargo until 2 pm Eastern Time on the date of publication. For more information, please contact onepress@plos.org.

Kind regards,

Philip A. Cola, Ph.D.

Academic Editor

PLOS One

Additional Editor Comments (optional):

Thank you for your careful revision of the manuscript and your thorough response to both the editorial and reviewer comments. The revisions have substantially improved the transparency of the study, particularly through the expanded description of the educational programme and study context, the clearer explanation of the sampling strategy and participant characteristics, and the addition of a dedicated section addressing trustworthiness and qualitative reporting standards.

The discussion and limitations have also been strengthened through a more balanced consideration of reflexivity, transferability, and methodological rigor. These revisions improve the overall clarity and credibility of the manuscript while maintaining the contribution of the work to the literature on person-centred leadership development in health and social care.

I appreciate the thoughtful manner in which the authors addressed the requested revisions and am pleased to recommend the manuscript for publication at this time.
